# Which positive factors determine the GP satisfaction in clinical practice? A systematic literature review

**DOI:** 10.1186/s12875-016-0524-x

**Published:** 2016-09-13

**Authors:** B. Le Floch, H. Bastiaens, J. Y. Le Reste, H. Lingner, R. D. Hoffman, S. Czachowski, R. Assenova, T. H. Koskela, Z. Klemenc-Ketis, P. Nabbe, A. Sowinska, T. Montier, L. Peremans

**Affiliations:** 1 ERCR SPURBO, Department of General Practice, Université de Bretagne Occidentale, Faculté de Médecine et des Sciences de la Santé, 22, avenue Camille Desmoulins CS 93837 29238, Brest, CEDEX 3 France; 2 Department of Primary and Interdisciplinary Care, Faculty of Medicine and Health Sciences, University of Antwerp, Antwerp, Belgium; 3 Centre for Public Health and Healthcare, Hannover Medical School, Hannover, Germany; 4 Department of Family Medicine, Tel Aviv University, Tel Aviv, Israel; 5 University Nicolaus Copernicus, Torun, Poland; 6 Department of General Practice, Medical University of Plovdiv, Faculty of Medicine, Plovdiv, Bulgaria; 7 Department of General Practice, University of Tampere, Tampere, Finland; 8 Department of Family Medicine, University of Ljubljana, Faculty of Medicine, Ljubljana, Slovenia; 9 Department of Family Medicine, University of Maribor, Faculty of Medicine, Maribor, Slovenia; 10 Department of English, Nicolaus Copernicus University, Torun, Poland; 11 Unité INSERM 1078, SFR 148 ScInBioS, Université Européenne de Bretagne, Faculté de Médecine et des Sciences de la Santé, Brest, France; 12 Department of Nursing and Midwifery, Faculty of Medicine and Health Sciences, Universiteit Antwerp, Antwerp, Belgium; 13 Mental Health and Wellbeing Research Group, Vrije Universiteit Brussel, Ixelles, Belgium

**Keywords:** Adult, Career choice, Career mobility, Family practice, General practitioners, Health care system, Humans, Job satisfaction, Physician, Primary health care

## Abstract

**Background:**

Looking at what makes General Practitioners (GPs) happy in their profession, may be important in increasing the GP workforce in the future. The European General Practice Research Network (EGPRN) created a research team (eight national groups) in order to clarify the factors involved in GP job satisfaction throughout Europe. The first step of this study was a literature review to explore how the satisfaction of GPs had been studied before. The research question was “Which factors are related to GP satisfaction in Clinical Practice?”

**Methods:**

Systematic literature review according to the PRISMA statement. The databases searched were Pubmed, Embase and Cochrane. All articles were identified, screened and included by two separate research teams, according to inclusion or exclusion criteria. Then, a qualitative appraisal was undertaken. Next, a thematic analysis process was undertaken to capture any issue relevant to the research question.

**Results:**

The number of records screened was 458. One hundred four were eligible. Finally, 17 articles were included. The data revealed 13 subthemes, which were grouped into three major themes for GP satisfaction. First there were general profession-related themes, applicable to many professions. A second group of issues related specifically to a GP setting. Finally, a third group was related to professional life and personal issues.

**Conclusions:**

A number of factors leading to GP job satisfaction, exist in literature They should be used by policy makers within Europe to increase the GP workforce. The research team needs to undertake qualitative studies to confirm or enhance those results.

**Electronic supplementary material:**

The online version of this article (doi:10.1186/s12875-016-0524-x) contains supplementary material, which is available to authorized users.

## Background

The international World Organization of National Colleges, Academies and Academic Associations of General Practitioners/Family Physicians (WONCA) definition of General Practice (GP) was established in 2002. It emphasised the specific, important and complex role of GP to ensure quality of care for the whole population [1, 2]. The World Health Organisation (WHO) stressed the central role of the GP, especially in European health care systems, having the same goal as WONCA [3]. However, the WHO also pointed out the recurrent problem of the low appeal of General Practice throughout Europe [4].

Health policy makers, aware of the problem of a decreasing GP workforce, have tried to change national policies in most European countries in order to strengthen General Practice. However, most of these policies addressed the negative aspects described in research, since most of the current research has focussed on negative aspects. For exemple they understood the difficulties arising from GPs’ burn out but where unable to change it, concentrating on working hours and work structure. By doing so they reduced the appeal of the profession instead of enhancing it [5–8]. Focusing positively on factors of satisfaction keeping General Practitioners (GPs) in their profession, may increase the likelihood of adequate GP provision in the future. This was the first research hypothesis we wanted to address in this study.

The European General Practitioner Research Network (EGPRN) is a network for research in General Practice that has designed a research agenda. One item on this agenda is the design of research exploring satisfaction among GPs across Europe. Consequently, the EGPRN decided to initialise a research into the satisfaction among GPs throughout Europe [9]. Another hypothesis of the group was that the feminisation of the GP workforce could have an influence on the future GP numbers. Therefore we wanted to to pay attention at WOmen, Men, and MANPOWER in this study which was consequently named “WoManPower”. This article is the first step in this research, aiming at systematically studying the literature to identify what is already available in terms of GP profession satisfaction factors.

## Methods

The EGPRN research group performed a systematic literature review with the focus on the GP job satisfaction. “Job Satisfaction” is a MESH term (Medical Subject Headings) which is defined by ‘a personal satisfaction relative to the work situation’. It was introduced in 1976 in the US national health library.

The review protocol was developed according to the PRISMA guidelines [10, 11]. Four researchers performed the entire process. The protocol was validated by the whole European team consisting of GP representatives from the following countries: Belgium, Bulgaria, Finland, France, Germany, Israel, Poland and Slovenia.

### Search strategy and inclusion criteria

Relevant studies were identified by systematic research in the databases Pubmed, Embase and Cochrane. The search was limited to articles published between 1 January, 2000 and 31 December, 2014. The year 2000 was set as the search start year as a turning point for the vision of GP with international work being initiated on its definition (and the WONCA definition being introduced in 2002) [1]. Practice and work organisation in general practice have been changed in many countries in the past decade, which might have a strong influence on job satisfaction. Four researches worked independantely with a merging of their results at each search and inclusion steps.

The database-specific search included the following algorithm for Pubmed: ((“Family Practice” [Majr] OR “General Practitioners” [Majr] OR “Physicians, Family” [Majr]) AND (“Career Choice” [Majr] OR “Career Mobility” [Majr])) AND hasabstract[text] AND (“2000/01/01” [PDAT]: “2014/12/31” [PDAT]) and ((“Family Practice” [Majr] OR “General Practitioners” [Majr]) OR “Physicians, Family” [Majr]) AND “Job satisfaction” [Majr] AND hasabstract[text] AND (“2000/01/01”[PDAT]: “2014/12/31”[PDAT]). Those equations were adapted for Cochrane and Embase according to their own specifications.

“Job satisfaction” was chosen by the research team because it was the best possible MESH term to describe positive factors at work. The use of a MESH term was efficient, because it included all possible synonyms.

In addition to the database search, grey literature was identified by the EGPRN’s national representatives. National teams (eight countries) were asked to provide grey literature citations, using the same keywords or known from experience. These were assessed by the national representatives and included in the review by consensus. Finally the bibliographies of all the included articles were checked in order to find additional references [11].

### Articles screening

Four independent researchers screened eligible articles based on title and abstract. Articles were considered relevant if the main focus was on positive factors related to General Practice. Only publications in English, Dutch, German and French were included. Abstracts were excluded if they were not reported in a structured way acoording to the IMRaD format (ie lacking a formal introduction, method result and discussion format), or if they only described a research protocol. Research articles performed in settings less relevant for the European context were also excluded such as studies undertaken in non-industrialised countries. The studies concerning other specialities were excluded because our review focused on GP. The review did not select the articles focusing on negative factors, or specific populations. Specific points raised by why students and trainees are attracted to General Practice were not explored in this review but are being addressed in additional on-going work.

### Articles eligibility and quality appraisal

Next, two research teams (French and Belgian) performed a full-text screening on the content and quality of all eligible articles. Because of the large heterogeneity in the types of studies, we assessed the quality of the articles with the criteria described in Table 1. To be included, the article had to score “yes” on every question. This quality appraisal form was adapted from the quality appraisal form of the CASP [12, 13].

**Table 1:** Quality appraisal

Did this article give an answer to the research question?	
Did the article focus clearly on the research question?	
Was the methodology appropriate?	
Was the recruitment appropriate?	
Do you believe the results? (Can it be due to chance, bias or confounding?)	

Differences of opinion at each step (screening and inclusion) were dealt with by discussion between the two researchers and, where there was a lack of consensus, the whole study group was consulted.

### Analysis of the data

Two researchers analysed the data from the included articles using a mixed method synthesis in which the findings of qualitative and quantitative studies were aggregated at the study level [14]. Results were pooled and anonymised at each step of the data extraction. A thematic analysis process was employed with the intention of capturing any issue relevant to the research question. These relevant issues were labelled and subsequently organised into subthemes and overarching themes. For the qualitative studies, the relevant results were the issues described in the research. For quantitative questionnaire based studies on GP satisfaction with different issues related to GP, only the items for which the mean satisfaction score was 60 % or above were included. (Different scales were used, so we recalculated this as a percentage of the total).

## Results

### Selection of the articles

**Fig. 1:**
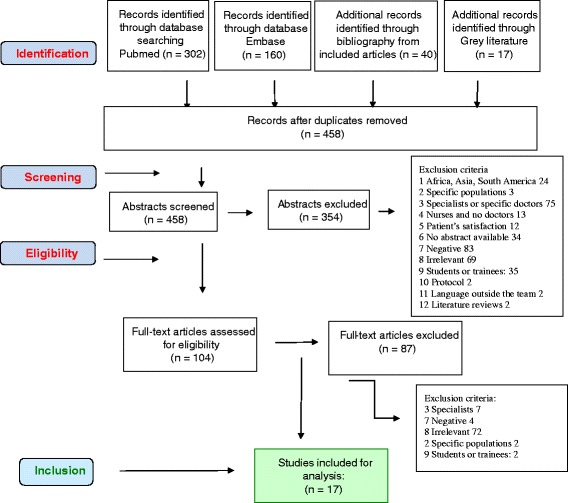
PRISMA diagram womanpower literature review GPs

The initial search in the different databases produced 458 different articles. Based on the abstracts, 104 full-text articles were eligible for further assessment and, ultimately, 17 articles were included in the review [15–31]. The full process is described in the PRISMA flow chart (Fig. 1).

All included studies were published in the English language. Six of the 17 studies were conducted in Canada, four in Australia, two in the USA, two in Germany, one in New Zealand, one in the United Kingdom and one in Switzerland.

Of these 17 selected publications, 11 were cross-sectional questionnaire studies; five used qualitative methods and one was a quantitative comparative study.

Additional file 1 provides an overview of the studies included in the review.

The research team collected 157 factors related to GP job satisfaction. Those factors were classified into 13 subthemes, which could be grouped into three overarching themes of issues supporting GPs satisfaction in their profession. First there are general profession related themes, applicable to many professions. A second group of issues is specific to a General Practice setting. Finally a third group is related to professional life and personal issues.

Those three groups of themes are described in Additional file 2.

### General profession related themes

Some topics found in the literature were not specific to General Practice, and could be found in other medical professions as well as in non-medical jobs. We described them under two subthemes: *“workload, income and the balance between them”* and *“getting responsibility and recognition for your work”.*

#### Workload, income and the balance between them

Working long hours requires sacrifices from GPs and their families. This topic was well explored in the literature. The topic of workload was studied in 13 of the 17 articles. The number of hours worked per week, including time spent on paperwork was not studied in the selected articles. Studies only looked at satisfaction related to working hours [23]. Many articles studied both income and workload. The main research question of these articles did not focus on satisfaction, but in comparing satisfaction with workload balance, related to gender or practice environment. We kept the results on income and workload balance as positive topics, but there was considerable variability among the different studies. What constituted a manageable workload and income balance depended on the individual lifestyle or gender of each GP. The question of gender was studied, for example in an Australian study: *“Female GPs were more satisfied than male GPs with their work-life balance”* [25]. There was no overall agreement on the concept of work-life balance. One important factor emerged: that of individual perception. “*Flexibility in work hours is a factor which increased GP satisfaction”* [18]. “*The freedom to choose the workload was important for job satisfaction”* [25].

The payment system had an effect on GP satisfaction about their income. GPs like to be involved in their payment method. In a Canadian study, “*rural physicians preferred the fee-for-service method, while urban physicians supported blended or fixed payment schemes”* [22]. Overall, “*GPs working in a rural area were more satisfied with their income than urban GPs”* [16, 19].

#### Responsibilities and recognition for quality of work

Having the opportunity to take responsibility, having a positive self-image and recognition for work undertaken were seen as important prerequisites for being satisfied with the profession. Being part of the community was another important satisfaction issue [15, 16, 19, 21, 22, 26]. “*GPs in smaller communities were slightly more satisfied with the amount of responsibility they had and recognition they received for good work”* [19]. A qualitative study, conducted in Nebraska, highlighted the GPs’ relationship with the social community: “*You are a very important part of the community and your opinion is listened to”* [26].

### Specific GP satisfaction factors

We grouped the specific GP related factors into nine sub–themes described below. Some of these aspects of satisfaction could be found in other professions, but these factors are more specific for the medical profession.

Successful medical management of patients and the subsequent feelings of being competent were associated with a higher degree of satisfaction. In a Scottish qualitative study, GPs derived the greatest satisfaction from consultations where GPs perceived that they personally had contributed to a successful outcome for the patient [27].

Freedom to choose the workplace and work organisation was one of the most relevant topics found in the literature, addressed in 13 articles. GPs wanted to have the freedom to choose their work method [16, 19, 22], their payment method [22], and have flexible working hours [18]. GPs need this freedom in order to have job satisfaction [24]. In a qualitative study, an American GP said: *“It’s a way of having independence and doing what I want”* [26].

Vocational choice; being able to be the kind of doctor you want to be: is an important sub-theme in being satisfied with your work as a GP. Six studies stated that the GP’s own personality and personal values played an important role. Values can differ between GPs. If their job were fully compatible with their temperament and personal values, then GPs were more satisfied. Some chose to be a “*traditional family doctor*” [21]. The mission for most GPs was to help people. “*The goal of practice is to meet people’s needs, take care of them and do the best you can*” [26].

The theme of GPs’ physical health, gender or age were assessed in the literature. A good health is associated with GP satisfaction. Not smoking and not being overweight is correlated with greater GP satisfaction. “*Older age, being female and having good health behavior has a positive effect on job satisfaction*” [16].

Intellectual stimulation to use abilities; continual professional development: This is one of the most studied aspects of satisfaction. Twelve articles in the review refered to the intellectuel aspect of the profession as a positive factor. GPs appreciated the intellectual challenge, and necessary skills that their GP work offered them. “*The intellectual and practical challenge to solve and assist with people’s medical problems”* [25]. “Doing a wider range of procedures was associated with higher overall job satisfaction” [24].

#### Relationship with patients and their families

Another group of potentially positive factors was related to the GP and his/her patient contacts and relationships. An efficient doctor-patient relationship was also considered an important factor in GP job satisfaction [17].

#### Relationship with other professionals

GPs need to work with colleagues. This topic was found in seven articles. They emphasised the importance of relationships with other specialities and hospitals [19, 22].

#### Variety in clinical practice; other professional challenges

^*«*^*General Practice is normally the point of first medical contact within the health care system, providing open and unlimited access to its users, dealing with all health problems regardless of the age, sex, or any other characteristic of the person concerned*^*»*^ [1]. The opportunity to deal with a variety of medical problems and patients was a positive aspect, even in rural areas [15, 16, 19, 21, 22].

Teaching and academic responsibilities was a final positive sub-theme related to GP practice. Positive GPs wished to communicate their work and skills. Eight articles studied the teaching role of GPs. Academic responsibilities gave GPs positive stimulation. *“Teaching, sharing knowledge and experience, and mentoring”* [23] *“Factors that contribute to career satisfaction for physicians include teaching and research”* [22]. As teaching is a factor for GP satisfaction, internships in General Practice were seen as extremely important in attracting students to General Practice [15]. This was an important influence on career choice for young students [31].

### Professional and private life

Nine articles described the influence of community on GPs’ job satisfaction. The community was important, especially in rural areas: “*Rural medical education is important for students from a rural background with a desire to work in a rural practice”* [29].

An important factor influencing career choice was the influence of the family or domestic circumtances [31]. GPs’ families wanted access to community services and leisure facilities.

## Discussion

The main contribution of this literature review is a description of a broad range of factors that GPs consider satisfying in relation to their job.

In 2006, Van Ham et al. published a systematic literature review about job satisfaction among GPs that described both decreasing and increasing trends in decisive factors [32]. The review found 24 relevant citations. The main factors increasing job satisfaction were: work diversity, relationships and contact with colleagues and being involved in teaching medical students. Our study confirmed these and added positive elements that could be targeted and used to support GPs and keep them in the job.

First, there are general professional aspects that are relevant for General Practice. Our review specifically highlights the importance of a balance between workload and income. Workload and income issues were frequently discussed but individual perceptions on their balance differed, related to age and gender [18, 25] A good workload balance for the female GPs seems linked to a lower number of hours worked. A good income is important for every GP. However, the pitfall is that GPs with higher income rates were less satisfactied due to their heavy workload. The challenge is to find the right balance between them [16]. This theme is important for all professions and can be approached from a negative perspective when overwork leads to burn out, or in a positive light when the workload and income are appropriate. A good work-life balance reinforces GPs’ job satisfaction. Policy makers should not force GPs to change this personal choice of workload and income balance.

Second, specific factors related to General Practice are linked with satisfaction. Of great importance for GP satisfaction is the freedom to organise and manage their own work and to be able to be the kind of doctor they want to be. Providing a context where GPs can build, run and organise their practice in line with their personal values seems a better way of maintaining satisfaction among the GP workforce.

Next, the literature analysis highlights the importance of challenges such as intellectual stimulation [25], and being able to practise a wide number of procedures (23). The diversity in practice is the first characteristic of General Practice, according to the WONCA definition. The variety in General Practice is a positive aspect well studied in the literature, found in nine articles in our review. The opportunity to widen activities to teaching and doing academic work, was also found to contribute to a high level of job satisfaction for some GPs, along with feeling clinically competent [27]. With regard to the latter, being able to make a personal contribution to a patient’s health is another very relevant experience contributing to job satisfaction. Our systematic literature review has shown that a satisfied General Practitioner wants to be clinically competent.

« Person centered care » which links to one core competency of the WONCA [1] should also be taken into account as a key competency for being a happy and successful General Practitioner. Great attention should be paid to that competency in initial and on-going medical education.

The unique doctor-patient relationship, while feeling useful and being integrated into the community, makes satisfied GPs. Policy makers must keep in mind, when reorganising the professions in primary care, that the most attractive factor in the profession seems to be the unique doctor-patient relationship, along with the longitudinal care and diversity in the work, which are extremely attractive factors in the profession [17, 23, 25].

Finally, the systematic literature review found that extra professional factors were important to be a satisfied GP. These extra professional factors are those that are of importance for the GP and his/her family, such as strong social support, schools, leisure activities and a good quality of life in the living environment. A strong link with the community and social recognition were also important for increasing the GP workforce. Stakeholders and health system should take this aspect into consideration.

### Strengths and limitations of the study

One of the results of this literature review is a broad overview of the positive factors which keep GPs in their clinical practice. This comprehensive view on satisfaction factors cannot be found by reading the individual articles. Most research on GP satisfaction was undertaken in a particular context, with small numbers, and with a focused research question on specific aspects. Merging all those factors provides a broader perspective on the topic. This richness of data has been obtained using a broad selection of articles.

The major part of the literature focused heavily on the material working condition of GPs. These studies were almost exclusively based on surveys which used declarative questionnaires, such as the Warr-Cook-Wall job satisfaction scale, which is not specific to General Practice [33]. These questionnaires did not examine in depth the aspects GPs might find satisfying in their profession. The data from the qualitative studies were very relevant. Qualitative studies allow a deeper investigation into the specific aspects of General Practice. However their research question sometimes focused on a particular context.

Confounding factors or interpretation bias could come from differences between social health systems and linguistic understanding. This bias was limited by working with an international research team, containing GPs from diverse cultures, and regular users of different health systems. During the screening of the research, different ways to estimate GP satisfaction were found. Some of the studies asked, “Why are you satisfied?” or “Which policies would lead to an improvement in GP satisfaction?” The research group decided to keep the various question forms although this could have caused confusion bias.

### Implications for practice and research

These results are of interest because most of the policies which tried to increase the GP workforce were based on non-specific professional aspects. This literature review opens up new possibilities for increasing the GP workforce by referring to specific activities within General Practice.

The literature review draws up a description of the General Practitioner who is satisfied in his/her work. Satisfied GPs are professionals who can keep a reasonable workload balance, which provides sufficient income and who are free to organise their work and determine how they work. Satisfied GPs are sufficiently challenged in their work and feel competent and useful. They have opportunities to broaden their tasks such as being involved in teaching or academic work. They wish to preserve their own healthand value good relationships with their patients and other professionals and live in an environment/community which appeals to them and to their family. These results will be transmitted to stakeholders of each of the participating countries, to be used in discussions with policy makers on the future of General Practice. This literature review provides new opportunities to improve health systems in OECD countries by referring to the satisfaction factors. To improve the strength of health work, a policy must promote a balance between work and income for GPs. It must take advantage of the skills of GPs, give them responsibilities and strengthen their image in the society. Policy makers now have a broad view of which factors constitute job satisfaction for GPs and could integrate them into their policies to increase the workforce.

This literature review showed numerous factors related to job satisfaction among GPs which are not widely explored elsewhere. Qualitative surveys seem important in the identification of these factors. Therefore, as the next step, the research team is committed to looking at these factors using a qualitative approach, with interviews and focus group discussions with GPs.

## Conclusions

Satisfaction factors are available to increase the GP workforce. Some have never been integrated into health system policies, such as clinical teaching, role modelling, freedom in work management, freedom in organisation of their working environment, intellectual stimulation, person-centred care and effective medical management of patients. The importance of freedom in organisation and management of care should stimulate the interest of stakeholders. The importance of clinical teaching should be noted by stakeholders and teachers. Specific GP practice settings need to be used in initial medical education. This positivist approach could lead stakeholders to take decisions which are directed towards increasing the GP workforce.

## Additional files

Additional file 1: Studies included in the review. (PDF 54 kb) Additional file 2: Overview of the results of the review. (PDF 40 kb)

## Abbreviations

EGPRN: European general practice research network
GP: General practice
GPs: General practitioners
MESH: Medical subject headings
UBO: Université de Bretagne Occidentale, France
WHO: World Health Organisation
WONCA: World Organization of National Colleges, Academies and Academic Associations of General Practitioners/Family Physicians
