# Supplementary material for: Which positive factors determine the GP satisfaction in clinical practice? A systematic literature review
Source: BMC Fam Pract. 2016 Sep 13;17(1):133. doi: 10.1186/s12875-016-0524-x (PMC5020554; doi:10.1186/s12875-016-0524-x)
Supplement: Additional file 1: — Studies included in the review. (PDF 54 kb) [file 12875_2016_524_MOESM1_ESM.pdf]

Ref in texte	First author	Title	Year	Journal	Langage	Country	Aim of the study	Method
14	Meli DN	General practitioner teachers' job satisfaction and their medical students' wish to join the field : a correlational study	2014	BMC Fam Pract.	English	Switzerland	To determine if medical students' desire to become GPs is related to the job satisfaction of their teaching GPs and explore the factors tied to this job satisfaction.	Questionnaire
15	Goetz K	The influence of workload and health behavior on job satisfaction of general practitioners.	2013	Family Medicine	English	Germany	To identify influencing factors on job satisfaction with regard to general practitioners' (GPs) characteristics such as age, gender, health behavior, body mass index (BMI), and workload.	Questionnaire
16	Behmann M.	Job satisfaction among primary care physicians: Results of a survey	2012	Deutsches Arzteblatt	English	Germany	Job satisfaction among PCPs in Lower Saxony, a large federal state in Germany.	Questionnaire
17	Shrestha D	Aspects of work-life balance of Australian general practitioners: determinants and possible consequences	2011	Aust J Prim Health.	English	Australia	To investigate the extent, determinants and possible consequences of work-life balance of Australian GPs.	Questionnaire
18	McGrail MR	Professional satisfaction in general practice: does it vary by size of community?	2010	Med J Aust.	English	Australia	To investigate whether the level of professional satisfaction of Australian general practitioners varies according to community size and location.	Questionnaire
19	Noonan T	When should I do rural general practice? A qualitative study of job/life satisfaction of male rural GPs of differing ages in New Zealand.	2008	N Z Med J.	English	New Zealand	To investigate the perceived advantages and disadvantages of rural general practice at various stages of family life of male NZ-trained GPs.	SS Interviews
20	Geneau R	Primary care practice a la carte among GPs: using organizational diversity to increase job satisfaction.	2007	Fam Pract.	English	Canada	To elicit its different facets and to understand better how organizational factors affect it.	SS Interviews
21	Lepnurm R	Factors associated with career satisfaction among general practitioners in Canada	2007	Can J Rural Med.	English	Canada	To articulate models that explain career satisfaction among general practitioners (GPs) in these practice environments.	Questionnaire
22	Manca DP	Rewards and challenges of family practice: Web-based survey using the Delphi method	2007	Can Fam Physician.	English	Canada	To identify and describe the important rewards and challenges that affect family physicians in Alberta.	Delphi method
23	Rivet C	Hands on: is there an association between doing procedures and job satisfaction?	2007	Can Fam Physician.	English	Canada	To determine whether there is a relationship in family medicine between higher overall job satisfaction and doing a wider range of procedures.	Questionnaire
24	Walker KA	What keeps Melbourne GPs satisfied in their jobs?	2007	Aust Fam Physician.	English	Australia	To investigate strategies that maintain and improve Melbourne (Victoria) GP job satisfaction.	Questionnaire
25	Backer EL	The characteristics of successful family physicians in rural Nebraska: a qualitative study of physician interviews	2006	J Rural Health.	English	United states	To explore rural physicians ' unique characteristics affecting their decisions and satisfactions with practice in a rural area.	SS Interviews
26	Fairhurst K	What general practitioners find satisfying in their work: implications for health care system reform.	2006	Ann Fam Med.	English	Scotland	To explore general practitioners' satisfaction with their patient visits and the congruity between this satisfaction and new models of practice, such as those implicit in the new general medical services contract in the United Kingdom.	SS Interviews
27	Chan BT	Factors influencing family physicians to enter rural practice: does rural or urban background make a difference?	2005	Can Fam Physician	English	Canada	To examine where rural physicians grew up, when during their training they became interested in rural medicine	Questionnaire
28	Rourke JT	Relationship between practice location of Ontario family physicians and their rural background or amount of rural medical education experience.	2005	Can J Rural Med	English	Canada	To determine if there was a difference in rural background and rural medical education experience between practising rural physicians and practising urban physicians in Ontario.	Questionnaire
29	Carek PJ	Practice profiles, procedures, and personal rewards according to the sex of the physician.	2003	South Med J.	English	United states	To explore physician and practice characteristics according to sex.	Questionnaire
30	Shanley BC	Factors influencing career development of Australian general practitioners.	2002	Aust Fam Physician.	English	Australia	To evaluate factors influencing career experiences and career choices made by former general practitioner registrars and to ascertain the reasons for these career decisions.	Questionnaire
