# Supplementary material for: Which positive factors determine the GP satisfaction in clinical practice? A systematic literature review
Source: BMC Fam Pract. 2016 Sep 13;17(1):133. doi: 10.1186/s12875-016-0524-x (PMC5020554; doi:10.1186/s12875-016-0524-x)
Supplement: Additional file 2: — Overview of the results of the review. (PDF 40 kb) [file 12875_2016_524_MOESM2_ESM.pdf]

N° of the reference in the text	Articles First author, year	General professional theme		Specific GP's practice									Professional and private life		Number of code by article
		Workload balance and income	Responsibilities and recognition of quality of work	Successful medical management of patient Feelings of being competent	Freedom to choose workplace and organise work	Vocational choice; be able to be the doctor you want	Role of GP's personality, gender, physical health	Intellectual stimulation to use abilities; continual professional development	Relationship with patients and their families	Relationship with other professionals	Variety in clinical practice; other professional challenges	Teaching and academic responsibilities	Supporting influence of community	Influence of the family	
14	Meli DN; 2014	X	X		X			X		X	X				6
15	Goetz K, 2013	X	X		X		X			X	X				6
16	Behmann M.; 2012	X			X		X	X	X	X					6
17	Shrestha D, 2011	X			X		X						X	X	5
18	McGrail MR, 2010	X	X		X			X		X	X		X	X	8
19	Noonan T, 2008	X							X		X		X	X	5
20	Geneau R, 2007	X	X	X	X	X		X	X	X	X				9
21	Lepnurm R, 2007	X	X		X		X			X		X	X	X	8
22	Manca DP, 2007	X			X			X	X		X	X			6
23	Rivet C, 2007	X			X		X	X	X			X	X	X	8
24	Walker KA, 2007	X		X	X			X	X		X				6
25	Backer EL, 2006		X	X	X	X		X	X		X		X	X	9
26	Fairhurst K, 2006			X		X		X	X						4
27	Chan BT, 2005					X	X	X				X	X	X	6
28	Rourke JT, 2005				X							X	X	X	4
29	Carek PJ, 2005	X			X	X		X						X	5
30	Shanley BC, 2002	X		X		X	X	X			X	X	X	X	9
Number of studies on the topic		**13**	**6**	**5**	**13**	**6**	**7**	**12**	**8**	**6**	**9**	**6**	**9**	**10**	
